# Novel Porcine Getah Virus from Diarrheal Piglets in Jiangxi Province, China: Prevalence, Genome Sequence, and Pathogenicity

**DOI:** 10.3390/ani14202980

**Published:** 2024-10-16

**Authors:** Jianhui Lan, Mengtao Fang, Leilei Duan, Zhong Liu, Guanggao Wang, Qi Wu, Ke Fan, Dongyan Huang, Yu Ye, Gen Wan, Yuxin Tang, Deping Song

**Affiliations:** 1 Department of Preventive Veterinary Medicine, College of Animal Science and Technology, Jiangxi Agricultural University, Nanchang 330045, China; lanjh981008@sina.com (J.L.); fmt15797711231@126.com (M.F.); cycahha132@126.com (L.D.); 19970042875@163.com (Z.L.); guanggaowang@163.com (G.W.); wuqi3950@163.com (Q.W.); fanke1112@163.com (K.F.); huangdongyan@jxau.edu.cn (D.H.); yy6157832@163.com (Y.Y.); wg8138@163.com (G.W.); tang53ster@gmail.com (Y.T.); 2 Jiangxi Engineering Research Center for Animal Health Products, Jiangxi Agricultural University, Nanchang 330045, China

**Keywords:** swine, Getah virus, epidemiology, virus isolation, genomic sequencing, pathogenicity

## Abstract

Getah virus (GETV) is a mosquito-borne virus belonging to the genus *Alphavirus* in the family *Togaviridae*. Its infection poses an increasing threat to animals and public health in China. In this study, an epidemiological survey of GETV on 46 pig farms in Jiangxi Province, China, was performed; GETV isolation and characterization were carried out, including a complete sequence determination and phylogenetic analysis; and pathogenicity of the GETV was experimentally investigated by inoculating newborn piglets with the isolated GETV strain. Epidemiological studies conducted on the organs of infected pigs, aborted piglets, and the blood of aborted sows sampled from pig farms in Jiangxi Province, China, demonstrated that 44 out of the 46 pig farms were positive for GETV, which is a positivity rate of 95.65% (44/46). Of the 411 samples tested, 47.93% (197/411) were found positive for GETV. A GETV strain called GETV-JX-CHN-22 was obtained, which showed stable proliferation in Vero cells. One-step growth curve results showed that the GETV-JX-CHN-22-P7 (passage 7) isolate reached a peak titer of 10^8.3^ TCID_50_/mL at 24 hpi. An analysis of the whole-genome sequencing results showed that GETV-JX-CHN-22 (prototype) and GETV-JX-CHN-22-P7 shared nucleotide sequence similarities of 95.3% to 99.6% with 73 reference strains of GETV in GenBank. Genetic evolution analysis revealed that GETV-JX-CHN-22 and GETV-JX-CHN-22-P7 belonged to the GIII group, the same group members of most strains were reported in China. Animal inoculation experiments indicated that piglets exhibited typical symptoms and pathological changes of GETV infection after 24 h inoculation, which reproduced the pathogenicity of GETV field strain infections in piglets. To our knowledge, this study is the first report on the detection and isolation of porcine GETV associated with diarrhea from pig farms in Jiangxi Province, China. It is of great importance to study the infection spectrum, transmission mechanism, and public health significance of GETV. The results provide foundations for the genomic and biological (pathogenic) characteristics of the circulating GETV in Jiangxi Province, China.

## Simple Summary

Pigs are now facing an increasing number of emerging and re-emerging viral agents that can cause significant economic losses. Identifying and studying the pathogenicity of these viral agents helps to prevent and control the spread of diseases. Getah virus (GETV) is a neglected zoonotic virus that causes reproductive disorders in sows and diarrhea in newborn piglets. To date, reports on GETV in pigs have focused mainly on reproductive disorders. In this study, we found GETV was frequently detected in piglets associated with diarrhea during epidemiological surveillance in Jiangxi Province in China. Piglets infected with GETV manifested as diarrhea, which could lead to death in suckling piglets. A GETV strain was isolated and characterized. The complete genome, pathogenesis, and pathogenicity of the isolated strain was investigated. Clinical signs, gross lesions, and histological changes were observed in suckling piglets inoculated with the isolated GETV strain. The results of this study add to the knowledge of the epidemiology, viral genetics, pathogenicity, and pathogenicity of porcine GETV.

## 1. Introduction

Getah virus (GETV) is a neglected mosquito-borne arbovirus that taxonomically belongs to the *Alphavirus* genus and *Togaviridae* family [1]. The genome of GETV is a linear, positive-sense, single-stranded RNA with an approximate size of 11.5 kb. The viral genome encodes a 5′-cap, two large open reading frames (ORFs, ORF1, and ORF2), and a 3′-poly(A) tail. ORF1, which is located at the 5′-end of the viral genome, encodes four nonstructural proteins (nsP1 to nsP4). ORF2, which is situated at the 3′-end of the viral genome, encodes structural polyproteins that are transcribed into five structural proteins (C, E3, E2, 6K, and E1) [2].

GETV was first isolated from *Culex* sp. mosquitoes in Malaysia in 1955 and was first found in an epizootic infection in racehorses in Japan in 1978 [3]. In China, GETV was first isolated from wild *Culex* mosquitoes in Hainan Province in 1964 and has recently been detected in and/or isolated from other mosquito species, including *Aedex vexans*, *Armigeres obturbans*, *Armigeres subalbatus*, and *Anopheles sinensis* [4]. Currently, GETV is widely distributed in 12 countries across Asia [5,6,7,8], Europe [9], and Australia [10,11]. Sero-epizootiological studies have demonstrated that GETV has a broad host spectrum, with natural infections in mosquitoes and animals including pigs, horses, goats, cattle, boars, blue foxes, and humans [12,13,14,15,16,17], suggesting that the host range of the virus has expanded widely. Recent studies have shown that GETV was first reported to infect pigs in Japan in 1965 and then spread rapidly across Asian countries [18]. In South Korea, several outbreaks in pigs caused by GETV were reported from 2003 to 2017 [19]. In China, porcine GETV was first identified in Taiwan in 2002 and subsequently in the provinces of Henan, Hunan, Anhui, Sichuan, Guangdong, Guangxi, Hubei, Shanxi, Fujian, Shandong, Jiangsu, and Jiangxi [17,20,21].

As a multi-host pathogen, GETV was becoming more serious and posing a serious threat to animal safety and public health [22]. Now, GETV has been found to be pathogenic to horses, pigs, and cattle, causing reproductive disorders, fever, neurological symptoms, diarrhea, and death in mammals, resulting in large economic losses in the livestock industry [23,24,25]. In addition, GETV can also infect wild animals, such as foxes, in which it can cause fever, anorexia, depression, neurological symptoms, and even death, and it is also associated with fever, anorexia, and depression in wild boars [26,27]. Pigs infected with GETV are presented with abnormal estrus, abortions, stillbirths, and fetal mummification in sows, while newborn piglets experience diarrhea, hypothermia, edema, ataxia, and death [20]. In some cases, the infected piglets developed pyrexia and anorexia, followed by ataxia and tremor [28].

Since October 2022, over 1000 piglets have died suddenly or from diarrhea on a pig farm in Jiujiang City in Jiangxi Province. However, the causative agents, such as PEDV, PDCoV, TGEV, PoRV, and SADS-CoV, could not be identified in these pigs. Unexpectedly, the primers targeting porcine GETV were positively amplified in over 50% of samples from these diseased pigs. GETV is considered a potential pandemic because of its expanding range and host spectrum. As of now, there are no vaccines or therapeutics available for this virus. To better understand the epidemiology, genetic evolution, and pathogenicity of GETV, we initially investigated the epidemiology of GETV in Jiangxi Province, which is a significant pork production region in China. Subsequently, growth kinetics, whole-genome sequencing, and phylogenetic analyses were performed to define the characteristics of GETV circulating in the area. Finally, the pathogenicity of the representative strain isolated in this area was evaluated.

## 2. Materials and Methods

### 2.1. Ethical Approval and Consent to Participate

The Jiangxi Agricultural University Institutional Animal Care and Use Committee approved the animal use protocol for this study (protocol number JXAULL-2022-10-35). All the procedures were carried out in accordance with The Care and Use Guidelines of Experimental Animals established by the Ministry of Agriculture of China. The specimens utilized in this study were collected from diseased pigs.

### 2.2. Case Presentation and Sample Collection

From May 2022 to March 2024, a total of 411 samples were collected from seven cities (Nanchang, Yichun, Jiujiang, Shangrao, Yingtan, Fuzhou, and Ganzhou) in Jiangxi, China. All samples were collected from sick pigs with diarrhea or neurological symptoms in suckling and weaned pigs and abortions, stillbirths, mummified fetuses, and bleeding in sows (background information listed in Table S1). Of the samples, 76 blood samples were collected from sows with abortions; 246 feces/intestines were collected from diarrheal suckling piglets (N = 189), weaned piglets (N = 20), and sows (N = 37); and 89 lung/lymph nodes were collected from mummified fetuses (N = 26), stillbirths (N = 42), and weaned pigs with neurological symptoms (N = 21). The samples were immediately delivered to the laboratory and stored at −80 °C. Viral RNA and DNA were extracted using a viral RNA/DNA extraction kit ver. 5.0 (TaKaRa, Dalian, China). Viral cDNA was synthesized using the PrimeScript^™^ II 1st Strand cDNA Synthesis Kit (TaKaRa, Dalian, China) according to the manufacturer’s instructions. The cDNA and DNA were subjected to PCR to detect suspected viral pathogens. According to the clinical symptoms of these pigs, several associated pathogens were detected using PCR-based assays, including porcine epidemic diarrhea virus (PEDV), porcine reproductive and respiratory syndrome virus (PRRSV), porcine rotavirus (PoRV), transmissible gastroenteritis virus (TGEV), porcine deltacoronavirus (PDCoV), and GETV. The primers used are presented in Table S2.

### 2.3. Virus Isolation Purification and Titration

A strong positive sample from a diarrheal piglet was homogenized and centrifuged at 10,000× *g* for 10 min at 4 °C. The supernatant was then filtered through 0.22-μm filters, and the filtrate was inoculated onto monolayers of Vero 81 cells. The cells were blind-passaged until cytopathic effects (CPEs) were observed. Samples without CPEs after 5 blind passages were discarded. If typical CPEs were observed, the cells were harvested, frozen, and thawed twice and then used to inoculate Vero 81 cells for viral passages. The RT-PCR-targeted GETV E2 gene was used to confirm the presence of GETV in the first 5 passages. The amplicons were then purified, cloned, and sequenced by Sangon Biotech in Shanghai, China. Finally, they were subjected to BLAST searches for confirmation of GETV.

For virus cloning/purification, the positive viral passages were serially diluted twofold in DMEM and then seeded into the monolayers of Vero 81 cells in a six-well culture dish, with each dilution added into three wells. After 2 h of adsorption, the inoculum was removed. The cells were overlaid with a DMEM mixture containing 1% low melting point agarose (Sangon Biotech, Shanghai, China) and 2% FBS. They were then incubated at 37 °C and 5% CO_2_ for 48–72 h. After incubation, the cells were stained with 0.5% crystal violet and 4% formaldehyde for 30 min and observed for visible plaques. Individual plaques were picked and passaged onto Vero 81 cells for three rounds to purify the virus. The titer of the purified virus was determined using Vero 81 cells and expressed as the TCID_50_ according to the Reed–Muench method.

### 2.4. Virus Plaque Assay and Growth Curve

Viral plaque assays were performed using Vero-81 cells grown in six-well plates. Viral samples were serially 10-fold diluted in DMEM, and 200 µL of each dilution was inoculated onto monolayers of Vero-81 cells and incubated for 1 h. The cells were then overlaid with a mixture of methylcellulose and 2% FBS and incubated at 37 °C for 3–4 days in 5% CO_2_. After carefully removing the medium, cells were stained with 3–4 mL of staining solution, consisting of 1% crystal violet and 4% formaldehyde, for 30 min and observed for visible plaques.

A 10-fold gradient dilution of the GETV virus solution to be measured was prepared using a DMEM cell maintenance medium containing 2% FBS. The virus solution was diluted from 10^−1^ to 10^−10^ and inoculated into a monolayer of Vero-81 cells in 96-well plates, with a volume of 100 μL. Eight parallel wells were created for each dilution. The plates were then placed in the culture under conditions of 37 °C and 5% CO_2_. Cell lesions were observed and recorded daily for 4 consecutive days. The TCID_50_ was calculated using the Reed–Muench method.

Viral growth kinetics were determined using Vero-81 cells, as described previously. Briefly, monolayers of Vero-81 cells inoculated into six-well plates at a multiplicity of infection (MOI) of 0.5 were incubated at 37 °C for 1 h. After incubation, Vero-81 cells were rinsed twice with PBS and replaced with a maintenance medium. Vero-81 cell supernatants were harvested at 12, 24, 36, 48, 60, and 72 h, respectively, and frozen at −80 °C until use. Viral titers (TCID_50_) at each time interval were determined and calculated using the Reed–Muench method to generate a viral growth curve.

### 2.5. Indirect Immunofluorescence Assay (IFA)

GETV infection was observed using IFA as described by Li et al. [29]. Briefly, Vero 81 cell monolayers were inoculated with the GETV isolate at an MOI of 0.1 for 2 h and then cultured with DMEM with 2% FBS for 24 h. Then, the cells were fixed with 4% paraformaldehyde, incubated at room temperature for 30 min, and washed twice with PBS (0.01 M, pH = 7.2). Next, the cells were sealed with 5% skimmed milk, incubated at 37 °C for 1 h, and then washed twice with PBS. The cells were reacted with a 1:1000 diluted GETV-Cap polyclonal antibody (prepared in our laboratory) and incubated at 37 °C for 1 h. They were then inoculated with FITC-conjugated goat anti-mouse secondary antibody at 37 °C for 1 h. Finally, 4′,6-diamidino-2-phenylindole (DAPI, Beyotime, China) was added to stain the nucleus, and the cells were incubated for 15 min at room temperature and observed under a differential fluorescence microscope (Axio Vert.A1, Zeiss, Germany).

### 2.6. Complete Genome Sequencing, Sequence Alignments, and Phylogenetic Analysis

To amplify the complete genome of the novel GETV strain isolated, 17 pairs of primers (Table S3) were designed based on the conserved regions identified through a multiple alignment on the sequences of GETV strains from GenBank. Fragments were amplified under the following conditions: denaturation at 95 °C for 2 min, 35 cycles (95 °C for 15 s, 53–56 °C for 15 s, 72 °C for 45 s), followed by a final extension at 72 °C for 5 min. PCR products obtained were subjected to gel purification using a gel extraction kit (TaKaRa, Dalian, China), and afterward, were cloned into pMD18-T vectors (TaKaRa, Dalian, China) following the manufacturer’s protocols. Five positive clones of each amplicon were submitted to a commercial sequencing company (Sangon Biotech, Shanghai, China) for sequencing in both directions using the Sanger sequencing methodology. The 5′- and 3′-RACEs for determining the terminal sequences were performed using a 5′/3′SMARTer RACE kit (Clontech, Beijing, China) following the manufacturer’s instructions.

The sequence fragments were imported to SeqMan in DNAStar *Lasergene* v7.10 (DNAStar, Inc., Madison, WI, USA) for assembly and annotation. The assembled complete genome sequences were deposited into GenBank under accession numbers OQ968487 and OQ863732, corresponding to strains GETV-JX-CHN-22 and GETV-JX-CHN-22-P7. Reference sequences for GETV were retrieved from the GenBank database at the National Centre for Biotechnology Information (NCBI) (https://www.ncbi.nlm.nih.gov/). A summary of the background information of GETV used in this study is shown in Table S4. The nucleotide and deduced amino acid (aa) of entire genome sequences and deduced aa sequences of the Cap, E3, E2, 6K, and E1 genes from the downloaded gene sequences and the GETV sequences obtained in this study were aligned using multiple sequence alignment by log-expectation (MUSCLE) and manually adjusted in MEGA v11 [30]. Phylogenetic trees were constructed using the neighbor-joining method in MEGA v11 with a bootstrap of 1000 replicates.

### 2.7. Inoculation of Piglets and Pathogenicity Investigation

To observe the pathogenicity of the isolated GETV strain, GETV-JX-CHN-22-P7, six ten-day-old GETV-negative piglets were randomly divided into two groups, with three piglets in each group. Piglets in one group were intravenously inoculated with 1.0 × 10^6.5^ TCID_50_/mL of GETV strain GETV-JX-CHN-22-P7 in 2 mL DMEM for each piglet, while piglets in the negative control group were intravenously administered 2 mL of DMEM. Pigs were monitored for signs of infection, including body weight loss, body temperature, hunched posture, lethargy, diarrhea, dehydration, and death. Feces were collected every 12 h for virus shed determination.

### 2.8. Pathological Changes and Virus Distribution in Organs, Serum, and Feces

Four days after the challenge, all of the piglets were necropsied and examined, and the serum, cerebrum, heart, lungs, spleen, kidneys, liver, colonic lymph nodes, mesenteric lymph nodes, small intestines (duodenum, jejunum, and ileum), large intestines (cecum, colon, and rectum), and feces were collected for viral load evaluation using real-time RT-PCR. RNA extraction and RT were performed as described above in epidemiological surveillance. The primers targeting the E2 gene are listed in Table S2. The standard plasmid was constructed by inserting the targeted E2 gene sequence of GETV into the pMD-19T vector (TaKaRa, Japan). Real-time RT-PCR was performed on the ABI 7500 thermal cycler (Applied Biosystems, Foster City, CA, USA) with qPCR SYBR Green Master Mix (YEASEN, Shanghai, China). The amplification conditions were as follows: 95 °C for 5 min, then 40 cycles of 95 °C for 10 s, 60 °C for 30 s, and 72 °C for 30 s. In each assay, 10-fold dilutions of the standard plasmid (from 10^8^ to 10^2^) and the negative control (distilled water) were included. Each sample was assayed three times. The copies of GETV viral RNA were calculated based on the results for the standard plasmid.

Histological observations on the brain, liver, heart, spleen, and intestines were executed according to the previous methods in our lab [31]. Briefly, tissues were fixed in 4% paraformaldehyde for 48 h, dehydrated with 30%, 50%, 70%, 95%, and 100% ethanol after 48 h fixation in 4% paraformaldehyde, cleared in xylene, embedded in paraffin wax, and sectioned at a 4–6 µm thickness. After dewaxing in xylene and serially rehydrating with 100%, 95%, and 70% ethanol, tissue sections were stained with hematoxylin and eosin (HE, Sigma-Aldrich, Shanghai, China) and then examined using conventional light microscopy.

### 2.9. Statistics Analysis

Differences were determined using Student’s *t*-test or one-way analysis of variance (ANOVA) in SPSS software 25.0 (IBM Corporation, Armonk, NY, USA) and visualized using GraphPad Prism 8.4.3. A *p*-value of <0.05 was set as the statistically significant level, and the *p* values are indicated as follows: ns ≥ 0.05; * *p* < 0.05; ** *p* < 0.01; *** *p* < 0.001; and **** *p* < 0.0001.

## 3. Results

### 3.1. Pathogen Identification and Epidemiological Investigation of Porcine GETV

A total of 411 samples were collected from clinically diseased pigs from 46 pig farms in Jiangxi Province, China, and then tested for GETV and other related viruses using specific RT-PCR assays. Overall, 44 out of 46 pig farms were positive for GETV, which is a positive rate of 95.65% (44/46). The prevalence of GETV in samples was found to be 47.93% (197/411), which was the highest among the 411 samples compared to the prevalence of other viruses (refer to Table 1 and Supplementary Table S1). PDCoV was the second most prevalent virus tested in our surveillance, with a detection rate of 30.21% (55/182). PEDV, PRRSV, and PRoV were detected at moderate rates in samples, with positive rates of 26.25%, 6.76%, and 16.63%, respectively. In addition, some GETV-positive samples were coinfected with PRRSV, PDCoV, PEDV, and/or PRoV. The most common co-infection was PEDV and GETV, with an average positive co-infection rate of 18.70%. On average, dual infections of GETV and PRRSV, GETV and PDCoV, and GETV and PRoV occurred at rates of 3.75%, 5.49%, and 8.51%, respectively.

### 3.2. Isolation and Characterization of GETV

**Figure 1:**
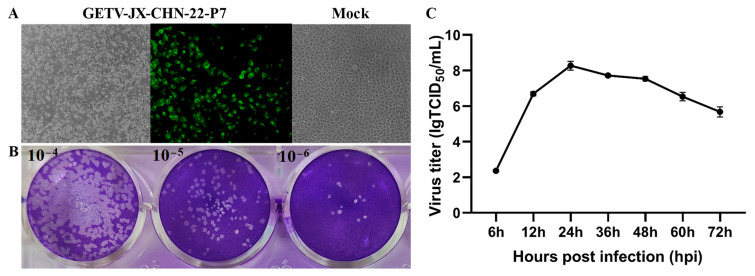
Biological characteristics of GETV-JX-CHN-22-P7. (**A**) Cytopathic effects (CPEs) in Vero-81 cells (100× magnification) at 36 hpi observed in a microscope and IFA. (**B**) Plaques formed in Vero-81 cells after inoculation with 10^−4^, 10^−5^, and 10^−6^ dilutions of GETV-JX-CHN-22-P7. (**C**) Growth situation of GETV-JX-CHN-22-P7 at a multiplicity of infection (MOI) of 0.01 on Vero-81 cells. Viral titers were determined as TCID_50_, and all values are presented as mean ± SD from three independent experiments.

A homogenized tissue sample from a diarrheal piglet that tested positive through RT-PCR was inoculated into Vero-81 cells for virus isolation. After two passages, CPEs were observed and were characterized by cell aggregation, shrinking, rounding up, and detachment in Vero-81 cells 36 h post-infection. An IFA analysis was conducted using an anti-GETV-Cap poly-antibody to confirm the isolation of the GETV strain (Figure 1C) (Figure 1A). The supernatants of each passage were confirmed to be GETV-positive by RT-PCR, and the GETV isolate was named GETV-JX-CHN-22. The isolate was purified through three rounds of plaque assay, and the purified isolate was named GETV-JX-CHN-22-P7. The plaques of GETV-JX-CHN-22-P7 exhibited a regular shape with clear visible edges, with an average diameter of 1.15 ± 0.35 mm (Figure 1B). To determine the growth kinetics, an 80% confluent cellular monolayer of Vero 81 cells was infected with GETV-JX-CHN-22-P7 at an MOI of 0.01 and harvested at 6, 12, 24, 36, 48, 60, and 72 hpi. GETV-JX-CHN-22-P7 exhibited a gradual increase from 6 hpi and reached a peak titer of 1 × 10^8.3^ TCID_50_/mL at 24 hpi (Figure 1C). Starting from 24 hpi, the viral titers decreased reaching a titer of ~10^6.0^ TCID_50_/mL at 72 hpi. The aforementioned results indicated that a strain of GETV had been successfully isolated.

### 3.3. Whole-Genome Sequencing and Phylogenetic Analysis

The complete genome sequences of GETV-JX-CHN-22 and GETV-JX-CHN-22-P7 are both 11,689 nt in length, excluding the poly(A) tail, and have a typical *Alphavirus* genome structure, including two large open reading frames (ORFs), ORF1 and ORF2, and short UTRs at the 5′ end (78 bp) and 3′ termini (401 bp). The genome sequences of GETV-JX-CHN-22 and GETV-JX-CHN-22-P7 were submitted to GenBank (accession no. OQ863732 and OQ968487). All 73 GETV strains in GenBank (as of August 11) were downloaded as reference strains for sequence comparison and phylogenetic analyses. The results showed that GETV-JX-CHN-22 and GETV-JX-CHN-22-P7 shared 95.1–99.3% sequence identities with these reference GETV strains at the nucleotide level and the highest identity (99.3%) with strains HNJZ-S2 (isolated in China, 2015) and GETV-YL (isolated in China, 2021). The GETV-JX-CHN-22-P7 strain showed the lowest homology with the original strain MM2021 (95.1%), while the highest homology with the GETV-JX-CHN-22 strain was isolated in this study (99.1%). Similarly, GETV-JX-CHN-22 exhibited a nucleotide homology range of 95.2% to 99.3% with other GETV strains at the complete genome level. The lowest homology was observed with the original strain MM2021 (95.2%), while the highest homology was observed with the HNJZ-S2 strain isolated from pigs in Henan Province in 2015 (99.3%) (Supplementary Table S5). Nucleotide (nt) mutations, which caused amino acid (aa) mutations, were observed between GETV-JX-CHN-22 and GETV-JX-CHN-22-P7 and the representative reference strains. Most of the nt mutations that caused aa mutations were found in NSP1~4, Cap, E2, and E1 (Supplementary Table S6).

**Figure 2:**
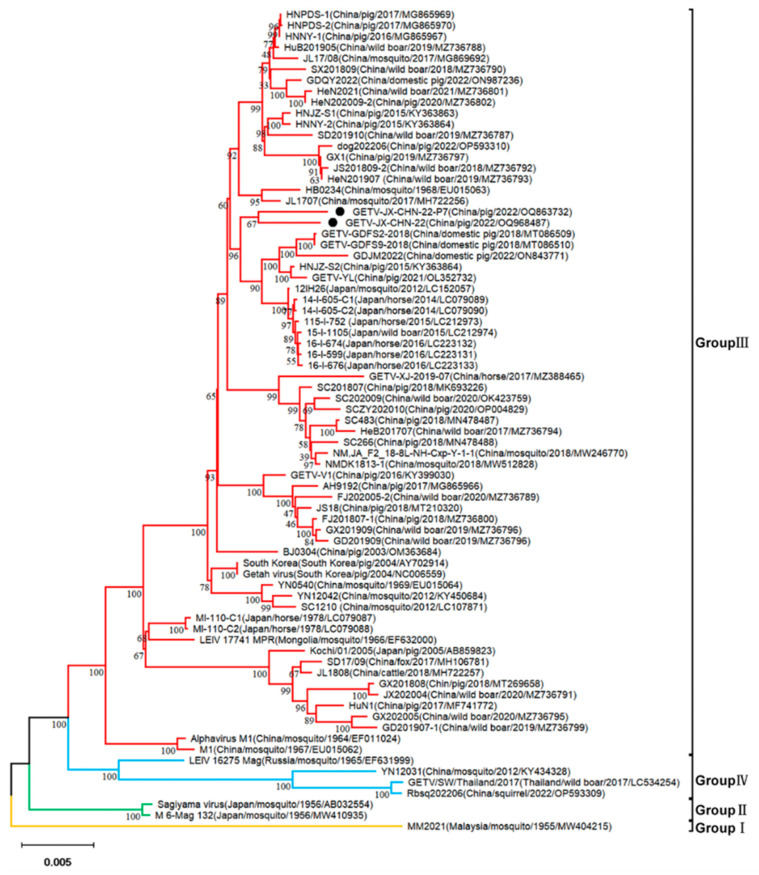
Phylogenetic analyses of complete genome (11,210 nt from ORF1 start codon to ORF2 stop codon, excluding 5′ and 3′ untranslated regions) of GETV. The phylogenetic tree was constructed using the p-distance-based neighbor-joining method with the MEGA v11 software. Bootstrap values (based on 1000 replicates) for each node are provided if they are >60%. The strain isolated in this study is labeled by a circle ●.

Based on the whole-genome sequences (from ORF1 start codon to ORF2 stop codon, 11,210 nt in length) and capsid and E2 gene sequences, phylogenetic trees were constructed using MEGA v11 with the p-distance-based neighbor-joining method (Figure 2). The results showed that the 75 strains can be divided into four evolutionary groups (Group I–Group IV). Group I contains a mosquito-borne strain, MM2021, that was isolated in Malaysia in 1955; Group II includes two mosquito-borne strains, Sagiyamavirus and M6-Mag132, that were isolated in Japan in 1956; Group IV includes a mosquito-borne strain, LEIV16275Mag, that was isolated in Russia in 1965, strain, GETV/SW/Thailand/2017, which was isolated from wild boars in Thailand in 2017, strain Rbsq202206, which was isolated from squirrels in Fujian Province, China, in 2022, and strain YN12031, which was isolated from mosquitoes in Yunnan Province, China, in 2012. Group III mostly consists of GETV strains from pigs, horses, mosquitoes, cattle, and dogs. Strain GETV-JX-CHN-22, which was isolated in this study, belongs to Group III, together with five Chinese GETV strains from pigs and eight Japanese GETV strains (six from horses, one from mosquitoes, and one from wild boars).

### 3.4. Clinical Manifestations and Necropsy Observations of Piglets Challenged with GETV-JX-CHN-22-P7

**Figure 3:**
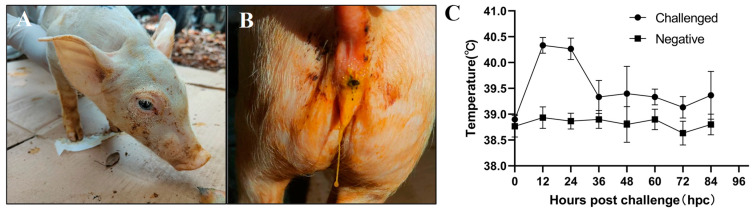
Clinical evaluation of piglets challenged with GETV strain GETV-JX-CHN-22-P7. (**A**) lethargy, (**B**) diarrhea, and (**C**) body temperature changes.

Two of the piglets intravenously injected with the GETV-JX-CHN-22-P7 strain displayed systemic tremors, lethargy, and anorexia at 12 hpi (Figure 3A) and mild diarrhea at 24 hpi (Figure 3B). At 60 hpi, piglets in the infected group showed various degrees of diarrhea and emaciation, along with symptoms such as decreased appetite and depression. The challenged piglets developed high fevers (exceeding 40 °C) at 12 hpi, which persisted for 12 h, and then gradually fell back and stayed at about 39.2 °C (Figure 3C). All piglets in the control groups were healthy and showed no clinical signs, and their body temperatures remained normal during the experiment.

**Figure 4:**
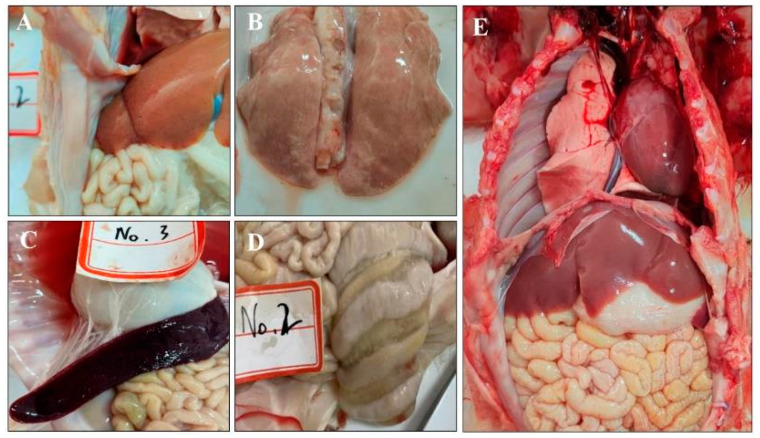
Gross lesions in piglets. Lesions in challenged piglets displayed petechial hemorrhages in the liver (**A**), pulmonary atrophy (**B**), splenic enlargement with hemorrhages (**C**), and colonic edema (**D**). The necropsies of the control group (**E**) revealed no significant abnormalities.

During the necropsies, gross lesions were observed with petechial hemorrhages in the liver (Figure 4A), pulmonary atrophy (Figure 4B), splenic enlargement with hemorrhage (Figure 4C), and colonic edema (Figure 4D). In contrast, no macroscopic lesions were observed in the pigs in the control group (Figure 4E).

### 3.5. Virus Distribution and Histopathological Examination

**Figure 5:**
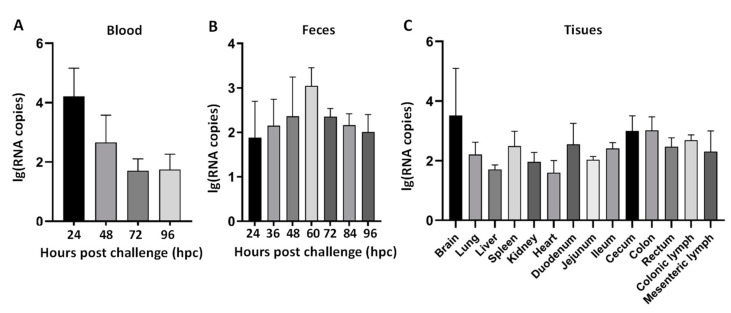
Virus load in blood (**A**), viral shedding in feces (**B**), and virus distribution in tissues at 96 hpi (**C**) of piglets challenged with GETV-JX-CHN-22-P7.

To further investigate the fecal virus shedding, viremia, and virus distributions in the GETV-challenged piglets, an RT-qPCR assay was introduced to detect GETV in blood, feces, and all tissues. The results of blood samples showed that GETV RNA copies reached a peak of 10^4.0^ copies per milliliter at 24 hpi, while in fecal samples, the peak was observed at 10^3.0^ copies per gram (copies/g) at 60 hpi (Figure 5A). The viral load demonstrated a trend of initially increasing and then decreasing, indicating continuous viral shedding throughout the course of infection in the piglets. The distribution of the GETV virus in different tissues showed that the viral load levels were relatively low in all tissues, ranging between 10^2.0^ and 10^3.5^ copies/g. The viral loads in the brain (average 10^3.5^ copies/g), cecum (average 10^3.0^ copies/g), and colon (average 10^3.0^ copies/g) were relatively higher, while the heart (average 10^1.6^ copies/g) had the lowest viral load (Figure 5C). No GETV RNA was detected in the negative control pigs during this study.

**Figure 6:**
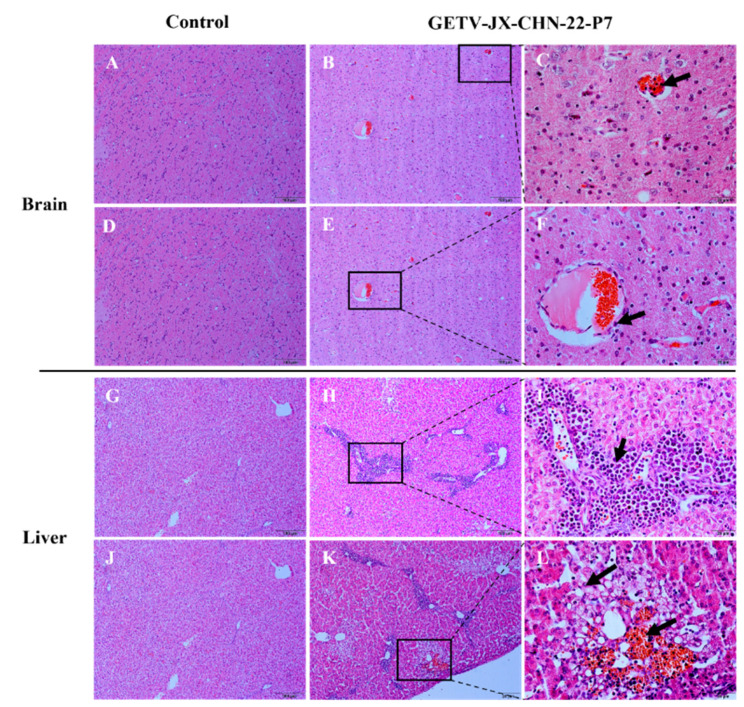
Clinical assessment of piglets challenged with GETV strain GETV-JX-CHN-22-P7. (**A**,**D**) Histologic view of brain tissues in control piglets. The scale bar indicates 100 µm. (**B**,**E**) Brain tissues showing congestion. (**C**,**F**) Higher magnification view of lesions boxed in panels B and E showing inflammatory cell infiltration in a vessel (arrows) and necrosis, along with mild neuronal degeneration. (**G**,**J**) Histopathologic examination of the brain and liver of control piglets revealed extensive inflammatory cell infiltration around the central veins, hepatocellular degeneration and necrosis with ballooning degeneration, and capillary rupture with hemorrhage (**H**,**I**,**K**,**L**). The scale bar in panels (**A**,**B**,**D**,**E**,**G**,**H**,**J**,**K**) indicates 100 µm, while the scale bar in panels (**C**,**F**,**I**,**L**) indicates 20 µm.

A microscopic examination revealed histopathologic lesions. The brain tissue showed lesions of non-purulent encephalitis, with mild neuronal degeneration and vascular inflammatory cell infiltration (Figure 6B,C,E,F). The liver tissue displayed a focal infiltration of lymphocytes and macrophages in the sinusoidal spaces, hepatocellular degeneration and necrosis, cytoplasmic loosening with ballooning degeneration, and inflammatory cell infiltration in the central veins, accompanied by hemorrhaging (Figure 6H,I,K,L).

**Figure 7:**
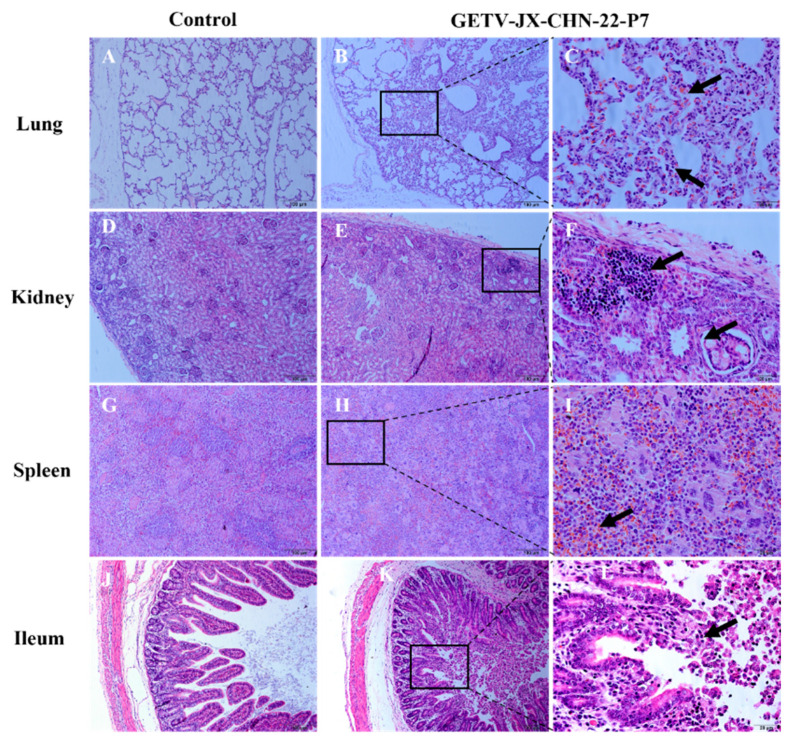
Clinical assessment of piglets challenged with GETV strain GETV-JX-CHN-22-P7. Histologic views of the lung, kidney, spleen, and ileum from control piglets (**A**,**D**,**G**,**J**) and challenged piglets (**B**,**E**,**H**,**K**). The scale bar indicates 100 µm. (**C**,**F**,**I**,**L**) Magnification of pathological changes in lung, kidney, spleen, and ileum in infected piglets, respectively. The scale bar in panels (**A**,**B**,**D**,**E**,**G**,**H**,**J**,**K**) indicates 100 µm, while the scale bar in panels (**C**,**F**,**I**,**L**) indicates 20 µm.

The lungs showed widened alveolar septa surrounding the bronchi, with desquamation of alveolar epithelial cells and thickening of the alveolar walls. The alveolar cavities showed hemorrhage, infiltration by macrophages, and neutrophils (Figure 7B,C). The kidneys showed focal inflammatory cell infiltration in the cortical layer, accompanied by erythrocyte extravasation, glomerular vasodilation, tubular epithelial cell swelling, and irregular lumina (Figure 7E,F). In the renal cortex, there was infiltration of inflammatory cells accompanied by erythrocyte extravasation, enlarged glomerular vessels, and swelling of the tubular epithelial cells, with irregular lumens. The spleen showed a reduction in white pulp volume, with visible hemorrhage and inflammatory cell infiltration, indicative of hemorrhagic splenitis (Figure 7H,I). In the ileum, extensive desquamation and fragmentation of the intestinal villi epithelial cells were observed (Figure 7K,L).

## 4. Discussion

GETV, an arthropod-borne zoonotic virus, was first isolated from *Culex gelidus* mosquitoes in Malaysia in 1955 [32,33]. Currently, GETV has a wide geographical presence, predominantly in the South Pacific (Northern Australia) and various Asian nations (China, South Korea, Japan, Thailand, the Philippines, Sri Lanka, Cambodia, and Vietnam). Since the inaugural isolation of the GETV-M1 strain in Hainan, China, in 1964, GETV has spread to several provinces. To date, China has identified over eighty strains of GETV, the majority of which have been isolated from mosquito vectors [34]. Meanwhile, GETV not only causes fever in humans but also infects a wide range of animals, including horses, foxes, and pigs, inducing disease manifestations [35]. Clinical signs vary among different animals and can include skin rashes, leg edema, fever, reproductive disorders, and fetal mortality [36,37]. In recent years, several outbreaks of GETV infections in swine have been documented across Southern China, with the most recent incident occurring in Heyuan City, Guangdong Province, in 2023 [20,38].

Notably, co-infection samples with multiple viruses were identified, with the highest co-infection rate of 18.70% (52/278) occurring with PEDV. However, the reports of co-infections involving GETV and enteric viruses remain limited. Due to the significant clinical and pathological similarities in terms of the manifestations caused by GETV and other porcine viruses, such as PRRSV, PEDV, and PRV, GETV infection is often underestimated in veterinary clinical practice, suggesting that more attention is needed. In this study, we successfully isolated a strain of GETV from a porcine intestinal sample in Jiangxi, thus reconfirming the presence of GETV in this region of China. Consistent with the results of the previous study that reported the isolation of strains of HuN1 and GETV-GDFS9-2018 [25,28], the GETV isolate GETV-JX-CHN-22-P7 replicated rapidly on Vero-81 cells and reached a peak titer of approximately 10^8.3^ TCID_50_/mL at 24 hpi.

Based on a multiple sequence alignment and phylogenetic analysis, the GETV-JX-CHN-22 isolated in this study and all the reference GETV strains share high nucleotide identities. Consistent with the previous study [39], GETV strains form four distinct genetic groups (Group I–Group IV). Group I included the original MM2021 strain isolated in 1964. Group II comprised two strains, the Sagiyama virus and M6-Mag132, which were isolated in Japan in 1956. Group IV consisted of the strains LEIV16275Mag, GETV/SW/Thailand/2017, YN12031, and the recently isolated Rbsq202206 from red-bellied squirrels in China. Meanwhile, Group III contained the majority of GETV strains, which were predominantly isolated in China [40]. The strains isolated in this study were close to the strains from China and belong to the Group III lineage, the present pandemic group, which is positioned on the same evolutionary branch. Additionally, GETV-JX-CHN-22 and GETV-JX-CHN-22-P7 shared 97.3% nucleotide sequence identity with the highly pathogenic HuN1 strain previously reported in Hunan Province, China, and 98.9% and 98.8%, respectively, with the pathogenic GDQY2022 strain recently documented in Guangdong Province [38]. Interestingly, the strains from this study were not located in the same evolutionary branch as the two strains. Instead, they exhibited a close phylogenetic relationship with the Henan HNJZ-S2 strain, as well as the Guangdong GETV-GDFS2-2018 and GETV-GDFS9-2018 strains.

Recent reports have shown a diversity of clinical signs in pig populations naturally infected with different strains of GETV [17]. In contrast, previous studies have shown discrepancies in the clinical manifestations between piglets experimentally infected with GETV and those naturally infected [41]. The pathogenicity of GETV strain Kanagawa was first confirmed in 5- to 18-day-old gnotobiotic pigs [42]. However, there is a paucity of research on the pathogenicity of GETV in natural versus experimental infections, with some studies lacking histopathological observations in infected pigs [43]. In this experiment, 10-day-old piglets inoculated with the GETV-JX-CHN-22-P7 isolate exhibited clinical signs of temporary fever, lethargy, anorexia, yellow diarrhea, and mild tremors. The symptoms are similar to those reported in previous studies of both natural and laboratory infections [23,24,25]. Necropsy results revealed varying degrees of lesions in all tissues of the inoculated piglets, except for the brain, heart, and kidneys. The lungs exhibited atrophy with grayish-white, island-like lesions; the liver displayed diffuse pinpoint hemorrhages; the spleen was swollen and congested; the ileum showed thinning of the intestinal wall; and the colon presented with edema. Notably, the lesions observed in the lungs, liver, and spleen have not been reported previously.

Tests of viral loads in various samples from the inoculated piglets revealed the presence of GETV in the blood, feces, and all organs examined. However, the overall levels of GETV were relatively low, which conflicts with recent findings reported by Pan et al., who reported higher levels of GETV in various tissues of naturally infected piglets [38]. Notably, their study did not test the virus in blood and feces. We detected a peak viral load of 10^4.0^ copies per milliliter in the blood of challenged piglets at 24 hpi. This finding aligns with previous observations of Kumanomido et al., who reported viremia in GETV-infected piglets at 1–2 dpi [41]. These results suggested that GETV may induce severe viremia during the initial stages of infection, with a subsequent gradual decline in viral levels in the blood as the infection progresses. The experimental results in this study demonstrated that GETV can be detected in the feces and blood of infected piglets and can persist over time, which is consistent with previous reports. This undoubtedly increases the potential for the transmission of GETV.

Histopathologically, the findings from piglets challenged with the GETV-JX-CHN-22-P7 strain in this study differed from piglets infected with the GETV HuN1 strain and the GDQY 2022 strain, as reported by Yang et al. [28] and Pan et al. [38]. Piglets infected with the HuN1 and GDQY 2022 strains have shown hepatocellular degeneration and necrosis, along with an increased number and aggregation of macrophages around the central veins and within the sinusoids. Likewise, piglets infected with the GETV-JX-CHN-22-P7 strain also showed hepatocellular cytoplasmic vacuolization presenting as ballooning degeneration accompanied by vascular rupture and hemorrhagic lesions in the liver. However, we observed that pigs infected with the GETV-JX-CHN-22-P7 strain exhibited focal inflammatory cell infiltration in the renal cortex accompanied by erythrocyte extravasation, swelling of the renal tubular epithelial cells, and irregular lumina. In the ileum, there was extensive desquamation and fragmentation of the villous epithelial cells. To our knowledge, the latter has not been reported previously. The findings of this study will undoubtedly advance our understanding of the clinical characteristics, pathogenicity, and epidemiological features of GETV

## 5. Conclusions

An epidemiological survey of GETV and common enteric viral pathogens was conducted based on samples collected from pig farms in Jiangxi Province, China, and the results demonstrated that the GETV-positive rate was 95.65% (44/46). Two GETV strains designated GETV-JX-CHN-22 (prototype) and GETV-JX-CHN-22-P7 (passage 7) were isolated and showed stable proliferation features in Vero cells. One-step growth curve results showed that the GETV-JX-CHN-22-P7 isolate reached a peak titer of 108.3 TCID_50_/mL at 24 hpi. A phylogenetic analysis revealed that GETV-JX-CHN-22 and GETV-JX-CHN-22-P7 belonged to Group III, the same group members of most strains reported in China. Animal challenge experiments indicated that piglets exhibited typical symptoms and pathological changes in GETV infection after 24 hpi. To our knowledge, this is the first report on the detection and isolation of porcine GETV from pig farms in Jiangxi Province, China. It is of great importance to study the infection spectrum, transmission mechanism, and public health significance of GETV. The results provide insights into the genomic and other biological characteristics of GETV circulating in Jiangxi Province, China.

## Figures and Tables

**Table 1:** Categorization of detection results on GETV and diarrhea-associated viruses of samples collected between 2022 and 2024.

Viruses	Detected Number	Positive Number	Positive Rate
Mono-virus			
GETV	411	197	47.95%
PRRSV	133	9	6.76%
PEDV	278	73	26.25%
PDCoV	182	55	30.21%
TGEV	182	0	0
PoRV	235	25	10.63%
Co-infection			
GETV + PRRSV	133	5	3.75%
GETV + PDCoV	182	10	5.49%
GETV + PEDV	278	52	18.70%
GETV + PoRV	235	20	8.51%

## Data Availability

The datasets presented in this study can be found in online repositories. The genome sequence of GETV-JX-CHN-22 and GETV-JX-CHN-22-P7 were submitted to GenBank under accession numbers OQ863732 and OQ968487, respectively.

## Supplementary Materials

The following supporting information can be downloaded at https://www.mdpi.com/article/10.3390/ani14202980/s1: Table S1: Background information and detailed detection results of samples collected in this study. Table S2: Primers used for diarrhea-associated viral pathogen detection in this study; Table S3: Primers used for amplifying the complete genome sequence of porcine GETV in this study; Table S4: Background information of reference strains of Getah virus; Table S5: Homology comparison between the GETV-JX-CHN-22-P7 isolate and reference GETV strains; Table S6. Comparison of amino acid sequence in nonstructural and structural proteins among the GETV strains isolated in this study and representative reference strains.
