# Supplementary material for: Novel Porcine Getah Virus from Diarrheal Piglets in Jiangxi Province, China: Prevalence, Genome Sequence, and Pathogenicity
Source: Animals (Basel). 2024 Oct 16;14(20):2980. doi: 10.3390/ani14202980 (PMC11503733; doi:10.3390/ani14202980)
Supplement: Supplementary file 1 [file animals-14-02980-s001.zip › Supplementary Tables 2-6.pdf]

Table S2. Primers used for diarrheal-associated viral pathogens detection in this study

Target	Sequence (5'-3')	Target gene	Product size
PEDV	Forward: GTATTGGTGGTGAGCGGAAT<br>Reverse: CCTGTTCCGCCATTCTATCA	ORF1	486 bp
PDCoV	Forward:CCAAACGCAACCCCAACAATCC<br>Reverse: CTTCTCAGTGTCTGCAGAGCCG	N	329 bp
TGEV	Forward: TATTTGTGGTTTTGGTTATAATGC<br>Reverse: GGCTGTTTGGTAACTAATTTGCCA	S	870 bp
PoRV	Forward: TATTCAAATATAAGTGATTTAATTCAAC<br>Reverse: TAATACCTGACAGCTTTCTTAATGC	VP6	298 bp
GETV	Forward: ACCGAAGAAGCCGAAGAA<br>Reverse: GCACTCRAGGTCATACTTG	Cap	316 bp
GETV	Forward: AAGTGGCAGTACACCTCCTC<br>Reverse: GTGGAGTTGGTCAGAGGGAA	E2	92 bp

Table S3. Primers used for amplifying the complete genome sequence of porcine GETV in this study

Name	Sequence	Position	Fragments size
GETV-1F0	ATGGCGGACGTGTGACATCA	0-20	932 bp
GETV-1R2	A CATAACCTTCGCATGACACC	912-932	
GETV-2F2	TTACCTTCCGTGTTTCACCTG	853-873	1080 bp
GETV-2R2	TGCCTGGAACTCACTCACTG	1913-1932	
GETV-3F2	GGTGTTGAAAAGCGAGAAGC	1773-1792	889 bp
GETV-3R2	TTGGTAGTGCAAGGTGGACA	2642-2661	
GETV-4F	GCGTGTCACTCGGGTACTTT	2446-2465	941 bp
GETV-4R	GCTGCGTATTTCCTGGCTAC	3367-3386	
GETV-5F	TGGTGCAAGTTCTTGAGACR	3128-3147	878 bp
GETV-5R	TACCGCTCTTCTGCCGTTAT	3986-4005	
GETV-6F	CTGGGAGGGGATTCAYTACA	3799-3818	829 bp
GETV-6R	CTAAGCAACTGTCCGGGTGT	4608-4627	
GETV-7F	GTACACCCGGACAGTTGCTT	4606-4625	718 bp
GETV-7R	TAGCGCGTATGTCTGTCGTC	5304-5323	
GETV-8F	TTCCATCGGAAACGACCTAC	5120-5139	865 bp
GETV-8R	TCATCCACCGATGGTGAGTA	5965-5984	
GETV-9F	GCAGGTACCAGTCGAGGAAR	5831-5850	735 bp
GETV-9R	GCCTTTCTTCGGTATGCTTG	6546-6565	
GETV-10F	ATACGGATCACCACCGAAAA	6382-6401	869 bp
GETV-10R	GGTTTCCCGAGTTTGAACAG	7231-7250	
GETV-11F	CGCCTTCATAGGTGATGACA	7029-7048	845 bp
GETV-11R	ATCTTCATGCACATGCGTTC	7854-7873	
GETV-12F	GCGAAGGCTAAGAAAAACGA	7755-7774	870 bp
GETV-12R	TATAGCCACCGGGCTGTARC	8605-8624	
GETV-13F	TGATGTGCGTCTTAGCCAAC	8344-8363	909 bp
GETV-13R	TAGGGACAAACGAGGAGGTG	9233-9252	
GETV-14F	CACCACCAGTAGCGACAAGA	9149-9168	894 bp
GETV-14R	AAGCCGTTCCTCTCAATGTG	10023-10042	
GETV-15F	GTATGCTGCCTGAAGAACYTG	9885-9905	803 bp
GETV-15R	CCAGAAGGGGTCTGTGTRTAA	10667-10687	
GETV-16F	GCATACTGCTTCTGCGAYAC	10245-10264	844 bp
GETV-16R	TGGCACTGCACACAGATACC	11069-11088	
GETV-17F	TGGATGCGTAATCAAGACCA	10742-10761	983 bp
GETV-R02	GTAAAATATTAAAAAAACAA	11670-11689	

Table S4. Background information of reference strains of Getah virus

GenBank accession No.	Strain Name	Host	Country	Year
AB032553	Sagiyama virus	*Mosquito*	Japan	1956
**AB859822**	**Kochi/01/2005**	***Sus scrofa***	**Japan**	**2005**
**AY702913**	**South Korea**	***Pig***	**South Korea**	**2004**
EF011023	Alphavirus M1	*Mosquito*	China	1956
EF631998	LEIV 16275 Mag	*Mosquito*	Russia	2000
EF631999	LEIV 17741 MPR	*Mosquito*	Mongolia	2000
EU015061	M1	*Mosquito*	China	1964
EU015062	HB0234	*Mosquito*	China	2002
EU015063	YN0540	*Mosquito*	China	2005
**KY363862**	**HNJZ-S1**	***Pig***	**China**	**2015**
**KY363863**	**HNJZ-S2**	***Pig***	**China**	**2015**
**KY399029**	**GETV-V1**	***Pig***	**China**	**2016**
KY434327	YN12031	*Mosquito*	China	2012
KY450683	YN12042	*Mosquito*	China	2012
LC079086	MI-110-C1	*Horse*	Japan	1978
LC079087	MI-110-C2	*Horse*	Japan	1978
LC079088	14-I-605-C1	*Horse*	Japan	2014
LC079089	14-I-605-C2	*Horse*	Japan	2014
LC107870	SC1210	*Mosquito*	China	2012
LC152056	12IH26	*Mosquito*	Japan	2012
LC212972	15-I-752	*Horse*	Japan	2015
**LC212973**	**15-I-1105**	***Sus scrofa***	**Japan**	**2015**
**LC223130**	**16-I-599**	***Pig***	**Japan**	**2016**
LC223131	16-I-674	*Horse*	Japan	2016
LC223132	16-I-676	*Horse*	Japan	2016
**LC534253**	**GETV/SW/Thailand/2017**	***Sus scrofa***	**Thailand**	**2017**
**MF741771**	**HuN1**	***pig***	**China**	**2017**
**MG865965**	**AH9192**	***Pig***	**China**	**2017**
**MG865966**	**HNNY-1**	***Pig***	**China**	**2016**
**MG865967**	**HNNY-2**	***Pig***	**China**	**2016**
**MG865968**	**HNPDS-1**	***Pig***	**China**	**2017**
**MG865969**	**HNPDS-2**	***Pig***	**China**	**2017**
MG869691	JL17/08	*Mosquito*	China	2017
MH106780	SD17/09	*Fox*	China	2017
MH722255	JL1707	*Mosquito*	China	2017
MH722256	JL1808	*Cattle*	China	2018
**MK693225**	**SC201807**	***Pig***	**China**	**2018**
**MN478486**	**SC483**	***Pig***	**China**	**2018**
**MN478487**	**SC266**	***Pig***	**China**	**2018**
**MT086508**	**GETV-GDFS2-2018**	***Pig***	**China**	**2018**
**MT086509**	**GETV-GDFS9-2018**	***Pig***	**China**	**2018**
**MT210319**	**JS18**	***Pig***	**China**	**2018**
**MT269657**	**GX201808**	***Pig***	**China**	**2018**
MW246769	NM, JA_F2_18-8L-N H-Cxp-Y-1-1	*Mosquito*	China	2018

MW404214	MM2021	*Mosquito*	Malaysia	1955
MW410934	M6-Mag132	*Mosquito*	Japan	1956
MW512827	NMDK1813-1	*Mosquito*	China	2018
MZ388464	GETV-XJ-2019-07	*Horse*	China	2017
**MZ736786**	**SD201910**	***Sus scrofa***	**China**	**2019**
**MZ736787**	**HuB201905**	***Sus scrofa***	**China**	**2019**
**MZ736788**	**FJ202005-2**	***Sus scrofa***	**China**	**2020**
**MZ736789**	**SX201809**	***Sus scrofa***	**China**	**2018**
**MZ736790**	**JX202004**	***Sus scrofa***	**China**	**2020**
**MZ736791**	**JS201809-2**	***Sus scrofa***	**China**	**2018**
**MZ736792**	**HeN201907**	***Sus scrofa***	**China**	**2019**
**MZ736793**	**HeB201707**	***Sus scrofa***	**China**	**2017**
**MZ736794**	**GX202005**	***Sus scrofa***	**China**	**2020**
**MZ736795**	**GX201909**	***Sus scrofa***	**China**	**2019**
**MZ736796**	**GX1**	***Pig***	**China**	**2019**
**MZ736797**	**GD201909**	***Sus scrofa***	**China**	**2019**
**MZ736798**	**GD201907-1**	***Sus scrofa***	**China**	**2019**
**MZ736799**	**FJ201807-1**	***Pig***	**China**	**2018**
**MZ736800**	**HeN2021**	***Sus scrofa***	**China**	**2021**
**MZ736801**	**HeN202009-2**	***Pig***	**China**	**2020**
**NC006558**	**Getah virus**	***Pig***	**South Korea**	**2004**
**OK423758**	**SC202009**	***Sus scrofa***	**China**	**2020**
**OL352731**	**GETV-YL**	***Pig***	**China**	**2021**
**OM363683**	**BJ0304**	***Sus scrofa***	**China**	**2021**
**ON843770**	**GDJM2022**	***Pig***	**China**	**2022**
**ON987235**	**GDQY2022**	***Pig***	**China**	**2022**
**OP004828**	**SCZY202010**	***Pig***	**China**	**2020**
OP593308	Rbsq202206	*Squirrel*	China	2022
OP593309	dog202206	*Dog*	China	2022

Table S5. Homology comparison between the GETV-JX-CHN-22-P7 isolate and reference GETV strains

Reference Strain	GETV GETV-JX-CHN-22-P7 (%)				
	Complete genome[a]	Non-structural polyprotein		Structural polyprotein	
	nt	nt	aa	nt	aa
GETV-JX-CHN-22	99.1	99.2	99.6	98.9	99.8
Sagiyama virus	97.1	97.2	99.1	96.8	98.7
Kochi/01/2005	97.6	97.6	99.5	97.7	99.6
South Korea	98.9	98.9	99.6	99.1	99.9
Alphavirus M1	97.8	97.8	99	97.8	99
LEIV 16275 Mag	97.4	97.3	99.3	97.4	99.5
LEIV 17741 MPR	98.3	98.2	99.3	98.6	100
M1	97.8	97.8	99	97.8	98.8
HB0234	98.9	98.9	99.2	98.8	99.6
YN0540	98.7	98.6	99.5	98.9	99.9
HNJZ-S1	98.9	98.9	99.4	99.1	100
HNJZ-S2	98.8	98.7	99.6	99.1	99.9
GETV-V1	98.8	98.8	99.5	98.8	99.8
YN12031	96.1	96.1	98.7	96.2	98.7
YN12042	98.6	98.4	99.4	98.8	99.8
MI-110-C1	98.3	98.2	99.5	98.6	99.9
MI-110-C2	98.4	98.2	99.5	98.6	100
14-I-605-C1	98.8	98.8	99.5	99	99.9
14-I-605-C2	98.8	98.8	99.5	99	99.9
SC1210	98.5	98.5	99.5	98.6	99.8
12IH26	98.9	98.8	99.6	99	99.9
15-I-752	98.8	98.8	99.4	99	99.9
15-I-1105	98.8	98.8	99.4	99	99.8
16-I-599	98.8	98.8	99.4	99	99.8
16-I-674	98.8	98.8	99.4	99	99.8
16-I-676	98.8	98.8	99.4	99	99.8
GETV/SW/Thailand/2017	95.9	96	98.9	95.7	98.6
HuN1	97.3	97.1	99.3	97.7	99.5
AH9192	98.5	98.5	99.2	98.4	99.4
HNNY-1	99	98.9	99.5	99.1	100
HNNY-2	98.9	98.9	99.5	99.1	100
HNPDS-1	99	98.9	99.5	99	100
HNPDS-2	99	98.9	99.5	99	100
JL17/08	98.9	98.9	99.5	99	99.8
SD17/09	97.4	97.4	99.4	97.5	99.5
JL1707	98.9	98.9	99.4	98.9	99.6
JL1808	97.5	97.5	99.4	97.6	99.6
SC201807	98.8	98.8	99.6	98.9	99.9
SC483	98.6	98.6	99.2	98.5	99.7
SC266	98.6	98.5	99.1	98.7	99.7
MM2021	95.1	95.2	98.7	95	98.2
GETV-GDFS2-2018	98.7	98.6	99.4	98.9	99.7

GETV-GDFS9-2018	98.7	98.6	99.4	98.9	99.7
JS18	98.6	98.7	99.6	98.5	99.5
GX201808	97.1	97	99.1	97.3	99.3
NM,JA_F2_18-8L-NH-Cxp-Y-1-1	98.7	98.7	99.6	98.6	99.8
M 6-Mag 132	97.1	97.2	99	97	99.1
NMDK1813-1	98.7	98.7	99.6	98.7	99.9
GETV-XJ-2019-07	98.3	98.3	99.3	98.4	99.8
SD201910	98.8	98.7	99.4	99.1	100
HuB201905	99	98.9	99.6	99.1	100
FJ202005-2	98.4	98.6	99.5	98.2	98.9
SX201809	98.8	98.7	99.5	99	100
JX202004	97	96.9	99.1	97.3	99.4
JS201809-2	98.7	98.7	99.5	98.8	99.7
HeN201907	98.8	98.7	99.6	98.8	99.7
HeB201707	98.4	98.4	99	98.5	99.7
GX202005	97.2	97.1	99	97.5	99
GX201909	98.5	98.6	99.6	98.5	99.5
GX1	98.8	98.7	99.6	98.8	99.7
GD201909	98.5	98.5	99.5	98.5	99.5
GD201907-1	97.1	97	98.9	97.5	99.4
FJ201807-1	98.6	98.6	99.6	98.5	99.5
HeN2021	98.8	98.7	99.4	98.9	99.8
HeN202009-2	98.8	98.7	99.4	99	100
Getah virus	98.9	98.9	99.6	99.1	99.9
SC202009	98.6	98.6	99.5	98.6	99.8
GETV-YL	98.8	98.7	99.6	98.9	99.8
BJ0304	98.8	98.8	99.4	98.8	99.8
GDJM2022	98.5	98.4	99.5	98.7	99.6
GDQY2022	98.8	98.8	99.6	99	100
SCZY202010	98.6	98.4	99.1	98.8	99.8
Rbsq202206	95.9	96	98.9	95.6	98.5
dog202206	98.7	98.7	99.6	98.6	99.4

[a]Complete genome (11,210 nt from ORF1 start codon to ORF2 stop codon, excluding 5′ and 3′ untranslated regions).

Table S6. Comparison of amino acid sequence in nonstructural and structural proteins among the GETV strains isolated in this study and representative reference strains

Viral Strains	Amino acid positions																
**NSP1**	73	207	245	427	435	455	462	465	475	478	498	521					
GETV-JX-CHN-22-P7	S	C	T	T	E	W	G	Q	T	G	V	A					
GETV-JX-CHN-22	S	C	T	T	E	W	G	Q	T	G	V	A					
HNJZ-S2	S	C	T	T	E	W	G	Q	T	G	V	A					
HNNY-2	S	C	T	T	E	W	G	Q	M	G	V	A					
HNPDS-1	S	C	T	T	E	W	G	R	M	G	V	A					
HeN202009-2	S	C	T	T	D	W	G	Q	M	G	V	A					
GETV-YL	S	C	T	T	E	W	G	Q	T	G	V	A					
M1	F	R	P	T	E	R	D	R	M	G	A	A					
MM2021	S	C	T	I	E	W	G	Q	M	S	A	T					
**NSP2**	84	244	299	455	542	614	644	667	757	783							
GETV-JX-CHN-22-P7	A	K	N	D	F	S	V	L	C	K							
GETV-JX-CHN-22	A	K	N	D	F	S	V	L	C	K							
HNJZ-S2	A	K	N	D	F	S	A	L	S	R							
HNNY-2	A	K	N	D	F	S	V	L	S	K							
HNPDS-1	A	K	N	D	S	S	V	L	S	K							
HeN202009-2	A	K	K	D	F	S	V	L	S	K							
GETV-YL	K	K	N	D	F	S	A	L	S	K							
M1	A	K	N	D	F	S	V	M	S	K							
MM2021	A	R	N	E	F	T	V	L	S	K							
**NSP3**	15	328	340	381	385	391	397	398	410	433	438	442	443	458	459	461	467
GETV-JX-CHN-22-P7	S	P	T	A	Q	V	V	T	E	R	T	P	S	T	S	P	E
GETV-JX-CHN-22	S	P	T	A	Q	E	V	A	A	R	A	P	L	T	S	P	E
HNJZ-S2	S	P	T	A	Q	E	V	A	A	R	A	P	L	T	S	P	E
HNNY-2	S	P	T	A	R	E	V	T	T	R	A	P	L	I	S	P	E
HNPDS-1	S	P	T	A	R	E	V	T	T	R	A	P	L	T	S	P	E
HeN202009-2	S	P	T	A	R	E	V	T	T	R	A	P	L	T	S	P	E
GETV-YL	S	P	T	A	Q	E	V	A	A	R	A	P	L	T	S	P	E
M1	G	P	T	A	Q	E	V	I	A	S	A	P	L	T	G	T	E
MM2021	G	L	A	V	Q	E	A	I	A	R	V	Q	L	I	S	T	G
**NSP4**	14	119	180	516	783												
GETV-JX-CHN-22-P7	Q	F	Q	A	S												

GETV-JX-CHN-22	Q	F	Q	A	S									
HNJZ-S2	Q	F	Q	T	S									
HNNY-2	Q	F	Q	T	S									
HNPDS-1	Q	F	Q	T	S									
HeN202009-2	H	F	Q	T	S									
GETV-YL	Q	F	Q	T	S									
M1	Q	F	Q	A	N									
MM2021	Q	D	R	T	N									
**Cap**														
GETV-JX-CHN-22-P7	34	69	75	77	79	156	183	206	34	69	75	77		
GETV-JX-CHN-22	V	P	Q	A	A	A	S	G	V	P	Q	A		
HNJZ-S2	V	P	K	A	A	A	S	G	V	P	K	A		
HNNY-2	V	P	K	A	A	A	S	G	V	P	K	A		
HNPDS-1	V	P	Q	A	A	A	S	G	V	P	Q	A		
HeN202009-2	V	P	Q	A	A	A	S	G	V	P	Q	A		
GETV-YL	V	P	Q	A	A	A	S	G	V	P	Q	A		
M1	V	P	K	A	A	A	S	G	V	P	K	A		
MM2021	-	P	Q	A	A	P	P	S	-	P	Q	A		
**E2**	27	90	102	109	122	205	248	314	323	368	374	386		
GETV-JX-CHN-22-P7	F	V	V	D	T	S	L	V	D	A	G	Y		
GETV-JX-CHN-22	F	V	V	D	T	S	S	V	D	A	G	C		
HNJZ-S2	F	V	V	D	T	S	L	V	D	A	G	Y		
HNNY-2	F	V	V	D	T	S	L	V	D	A	G	Y		
HNPDS-1	F	V	V	D	T	S	L	V	D	A	G	Y		
HeN202009-2	F	V	V	D	T	S	L	V	D	A	G	Y		
GETV-YL	F	V	V	D	T	S	L	V	D	A	G	Y		
M1	F	V	V	G	T	R	L	V	D	V	C	Y		
MM2021	S	T	A	D	I	S	L	A	E	A	G	Y		
**E1**	71	155	178	187	377	417	434	71	155	178	187	377	417	434
GETV-JX-CHN-22-P7	M	T	V	Q	M	L	V	M	T	V	Q	M	L	V
GETV-JX-CHN-22	M	T	V	Q	M	L	V	M	T	V	Q	M	L	V
HNJZ-S2	M	T	V	Q	M	L	V	M	T	V	Q	M	L	V
HNNY-2	M	T	V	Q	M	L	V	M	T	V	Q	M	L	V
HNPDS-1	M	T	V	Q	M	L	V	M	T	V	Q	M	L	V
HeN202009-2	M	T	V	Q	M	L	V	M	T	V	Q	M	L	V

GETV-YL	M	T	V	Q	T	L	V	M	T	V	Q	T	L	V
M1	M	T	V	L	M	F	G	M	T	V	L	M	F	G
MM2021	K	N	A	Q	T	L	V	K	N	A	Q	T	L	V
